# How general dentists could manage a patient with oral lichen planus

**DOI:** 10.4317/medoral.22368

**Published:** 2018-02-25

**Authors:** Jairo Robledo-Sierra, Isaäc van der Waal

**Affiliations:** 1 Department of Oral Medicine and Pathology, Institute of Odontology, Sahlgrenska Academy, University of Gothenburg, Gothenburg, Sweden; 2 Faculty of Dentistry, CES University, Medellin, Colombia; 3 Department of Oral and Maxillofacial Surgery/Pathology, VU University Medical Center/ Academic Centre for Dentistry Amsterdam (ACTA), Amsterdam, The Netherlands

## Abstract

**Background:**

The literature hardly contains information on how patients suffering from oral lichen planus could be managed by dentists.

**Material and Methods:**

Based on the limited available literature and particularly on the long-term clinical and histopathological experience of one of the authors, suggestions on how dentists could manage patients with oral lichen planus have been put forward. Results: In most cases, the dentist should be able to establish a correct diagnosis.

**Results:**

In most cases, the dentist should be able to establish a correct diagnosis. Occasionally, the dentist may call upon a specialist, usually an oral medicine specialist or an oral and maxillofacial surgeon for confirmation of the diagnosis, possibly a biopsy procedure, and management of the patient in case of severe symptoms. Proper patient information is of utmost importance in the management.

**Conclusions:**

General dentists can be expected to manage the majority of patients with oral lichen planus. Some patients may need to be referred for diagnostic purposes to a specialist; this is also the case for the rare patient with severe symptoms, possibly requiring systemic treatment.

** Key words:**Oral mucosal disease, oral lichen planus.

## Introduction

It is a challenge for general dentists to be able to clinically diagnose and manage the wide range of white or white-and-red lesions that may affect the oral mucosa. This is also true for oral manifestations of lichen planus. The purpose of this contribution is to provide some basic knowledge about oral lichen planus (OLP) and to suggest how dentists can manage their patients with this disease. The discussion is based on the available literature and on the long-term clinical and histopathological experience of the senior author (IvdW).

## What dentists should know about oral lichen planus and oral lichenoid lesions

2.1 Definition, epidemiology and etiology

Lichen planus (LP) is a chronic mucocutaneous disorder which may not only affect the skin and the oral mucosa but also the lips, nails, scalp, and other mucosal surfaces, including the vulvar and vaginal mucosa, glans penis, esophagus, and pharynx. Reported prevalence figures of OLP vary from less than 1% up to 4% (1). OLP mainly affects middle-aged people and is more common among women. Occurrence in children is rare (2).

Lichen planus is most likely caused by T- cell mediated autoimmunity. The exact etiopathogenesis is unknown (3). Psychological factors may play an important role (4). In some populations there is a significant, yet unexplained, association between lichen planus and hepatitis C virus (5). An association with thyroid diseases has also been reported in other populations (6). In addition, gallbladder disease was found to be present in nearly 20% of patients with OLP (7). These finding do not suggest that patients with OLP should be routinely screened for the presence of the aforementioned diseases, but rather are of interest from the etiopathogenic perspective.

Oral lichen planus-like lesions, usually referred to as lichenoid lesions or lichenoid reactions, may occasionally be caused by the use of systemic medication ( Table 1) (8,9). Prolonged, direct anatomic contact with a large amalgam restoration (“Contact lesion”) or other dental materials like composites and glass ionomers, may also provoke a lichenoid lesion in the oral mucosa. Furthermore, oral lesions of chronic graft-versus-host disease in patients with an allogenic stem cell transplantation may clinically resemble OLP. A classification of oral lichenoid tissue reactions (“interface mucositis”) is shown in  Table 2 (10).

**Table 1:**
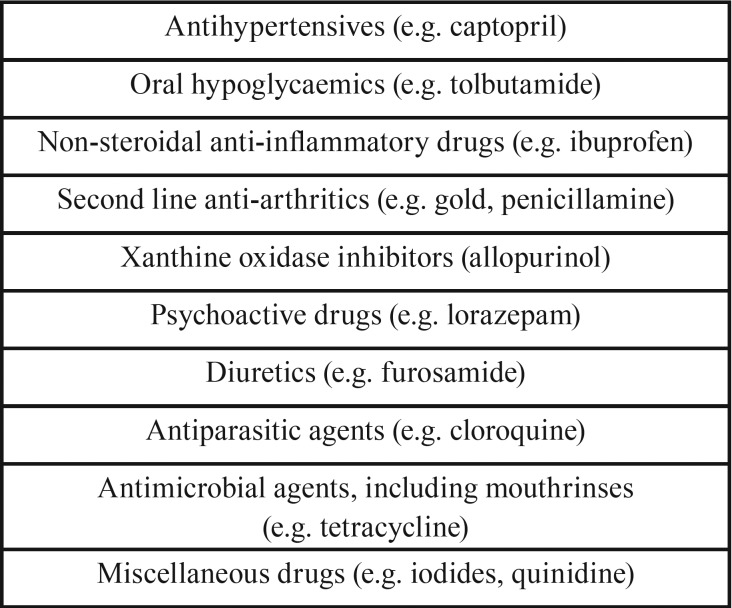
Some of the drugs that may cause lichenoid lesions (Rice and Hamburger, 2002 (8).

**Table 2:**
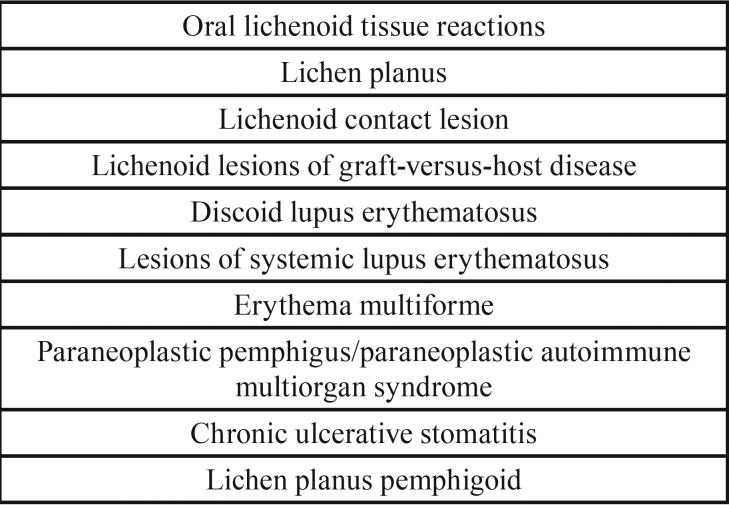
Classification of oral lichenoid tissue reactions according to Khudhur *et al.* (10).

2.2 Symptoms and clinical aspects

The course of OLP is characterized by periods of remission and exacerbation, where both the signs and the symptoms can last several weeks or even months. Symptoms may vary from a mild sense of roughness of the affected mucosa to itching and pain, particularly when eating spicy foods. In case of gingival involvement, bleeding on tooth brushing may be the major complaint. In case of visible, fiery redness of the gingiva, esthetic complaints are common.

OLP may show various clinical manifestations, such as reticular, annular, papular, erythematous, plaque- type, ulcerative and bullous appearance. Frequently, the adjectives “atrophic” and “erosive” are equivocally used in the clinical setting as synonyms for the erythematous and ulcerative types, respectively. Two or more clinical forms may occur simultaneously and may change over time. The buccal mucosa, the tongue, and the gingiva are the most often affected sites. There is nearly always a bilateral, more or less symmetrical distribution.

**Figure 1:**
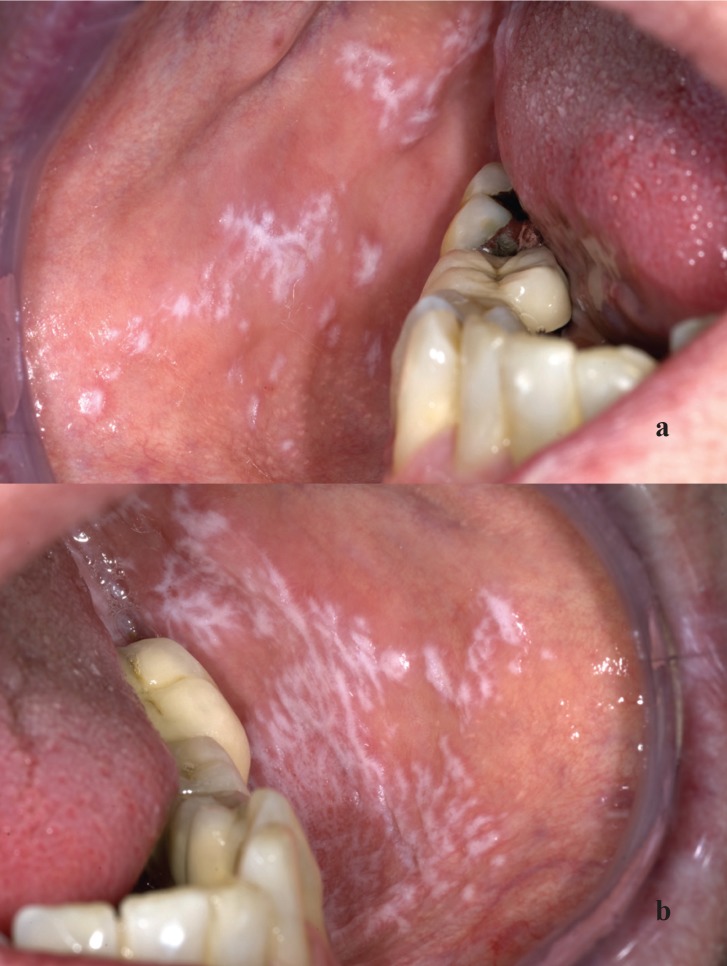
Typical presentation of oral lichen planus in an otherwise healthy patient (a and b); there is no need for a biopsy for diagnostic purposes.

In many cases the diagnosis of OLP can be made clinically, particularly when presenting in the reticular form (Fig. 1). At times, however, it may be difficult to distinguish the non-reticular types of OLP from the various subtypes of leukoplakia, erythroplakia, lichen sclerosus, lichen planus pemphigoides, lupus erythematosus, linear IgA disease, chronic ulcerative stomatitis, mucous membrane pemphigoid, and even the second stage of syphilis. In such cases a biopsy may be helpful for histopathologic examination and, if indicated, for immunohistochemical analysis.

2.3 Histopathologic aspects

The histopathological aspects of OLP are not always diagnostic on its own and there may be a discrepancy between the clinical diagnosis and the histopathologic diagnosis of OLP (11). Therefore, a close collaboration between the clinician and the pathologist should always be implemented.

2.4 Is oral lichen planus a premalignant disorder?

OLP is generally regarded as a potentially malignant disorder. The reported annual malignant transformation rate is approximately 1% (12). If a squamous cell carcinoma arises, it usually affects the tongue and not the sites affected by OLP (13). Other clinicians/researchers have not found any evidence of a potentially malignant behavior (14). Unfortunately, malignant transformation in OLP is neither predictable nor preventable in the individual patient.

2.5 Management

OLP may last for many years, if not lifelong. Unfortunately, there is no cure for OLP. Treatment can only be symptomatic and most commonly consists of topical or, occasionally, systemic administration of corticosteroids. The use of topical corticosteroids may enhance the risk of the development of candidiasis, which requires concomitant antimycotic therapy. There are also other drugs available, e.g. tacrolimus, thalidomide, topical aloe vera, topical retinoids, oral curcuminoids, and lysopine. The efficacy of all these drugs is rather questionable (15). A variety of other treatment modalities has been reported, such as surgical excision, laser evaporation, laser excision and photodynamic therapy. Such treatment modalities may be useful in persistent, localized areas of OLP involvement. It has been shown that plaque control improves the symptoms of gingival OLP (16). Stress management may be a useful part of the treatment protocol (17).

It is questionable whether long-term follow up reduces the risk of frank cancer development and whether such regime improves the survival rate of the patient (18,19).

## The role of the dentist/general practitioner

3.1 Diagnosis. It is a challenge for dentists/general practitioners to be able to clinically diagnose the wide range of white or white-and-red lesions that may occur in the oral cavity. Some dentists may always want to have their diagnosis confirmed by a biopsy in case of suspected OLP, e.g. for medicolegal reasons, even when presenting in the more or less classic, reticular form, while others may take a biopsy only in case of doubt about the diagnosis. Occasionally, it is the patient who insists of having a biopsy taken. Since most dentists have not been trained to perform a biopsy, they may call upon a specialist, usually an oral and maxillofacial surgeon or an oral medicine specialist.

In case of suspicion of a lichenoid lesion related to a dental restoration, the patient may be referred for allergy testing, e.g. to a dermatologist (20). In addition, referral for a biopsy should be considered before replacing the dental restoration, particularly in case of symptoms.

3.2 Examination for extraoral manifestations. Only in the presence of extraoral symptoms a referral to a dermatologist is indicated to exclude skin/genital involvement.

3.3 Evaluation of possible influence of medication taken for other diseases. The causal relationship between medications and OLP is difficult to assess since the effect of adjustment or replacement of the suspected agent may take several months or even years. Only in case of severe symptoms and/or large, rapidly progressing ulcerations evaluation of the possible influence of medications should be considered. For practical reasons, such patients should be referred to a specialist, e.g. an oral and maxillofacial surgeon or a stomatologist.

3.4 Treatment. In case of mild symptoms, topical use of corticosteroids can be prescribed by general dentists. There is no value in prescribing corticosteroids as a preventing therapy. If the patient develops candidiasis during or after the treatment with corticosteroids, the dentist may need the help of a specialist for guidance with the antimycotic medication. Optimization of oral hygiene is particularly recommended in patients with gingival involvement. In case of severe symptoms, where the use of systemic corticosteroids may be required, referral to a specialist is appropriate. In such event, the dentist should preferably retain the role of coordinator in the management of the patient (21). When stress management seems indicated, communication with the patient’s family doctor is advised.

Unavoidably, some patients want to be referred to a specialist even in case of mild symptoms.

3.5 Follow up because of the allegedly increased risk of oral cancer development. Although malignant transformation in OLP is neither predictable nor prevented in the individual patient, annual long-term follow up may be considered for reassurance of the patient. The dentist/general practitioner should be able to take care of such follow-up visits. In case of worsening of the clinical appearance, referral to a specialist is advised.

3.6 Patient information. The dentist/general practitioner should be able to provide patient information on OLP. Proper patient information is an important aspect in the management and should preferably be given in both verbal and written form. An example of a patient information in case of OLP is depicted in table 3.

## Conclusions

Dentists are expected to be familiar with the basic aspects of OLP. They are advised to refer any patient with an oral mucosal lesion that cannot be properly diagnosed on clinical grounds alone, as may be the case in lichen planus or lichenoid lesions to a specialist. In such event, a biopsy may or may not, be necessary. Dentists should be able to manage and follow up the majority of OLP patients with mild symptoms. Only in case of severe symptoms referral to a specialist is indicated. In any event, proper patient information is of utmost importance.
